# Effectiveness of hyaluronic acid vs. surgical and non-surgical methods for Papilla Reconstruction: a systematic review

**DOI:** 10.3389/fdmed.2026.1757417

**Published:** 2026-02-02

**Authors:** Amal Ghazi Jamjoom, Maha Ali Bakhshwain

**Affiliations:** 1 Department of Periodontology, Faculty of Dentistry, King Abdulaziz University, Jeddah, Saudi Arabia; 2 Bachelor of Dental Surgery (BDS), King Abdulaziz University Faculty of Dentistry, Jeddah, Saudi Arabia

**Keywords:** hyaluronic acid, papilla reconstruction, patient satisfaction, periodontal regeneration, tooth, anterior, treatment outcome

## Abstract

**Aims:**

Interdental papilla loss produces unaesthetic “black triangles” that impair function and patient satisfaction. While surgical and non-surgical techniques exist, hyaluronic acid (HA) injections have emerged as a minimally invasive option, but their comparative effectiveness and certainty of evidence remain unclear. This systematic review aimed to evaluate the clinical outcomes of injectable HA for interdental papilla reconstruction.

**Materials and methods:**

MEDLINE/PubMed and EMBASE were searched from inception through May 2025 for peer-reviewed randomized and non-randomized trials, cohort studies, or split-mouth designs in adults aged 18 years or older. Eligible studies compared injectable HA to surgical (e.g., connective tissue grafts) or non-surgical approaches (e.g., platelet-rich fibrin, saline placebo). The risk of bias was assessed using Cochrane RoB 2.0 and the ROBINS-I tool. Where appropriate, meta-analyses were performed, and heterogeneity was evaluated using the *I*^2^ statistic.

**Results:**

Three trials (*n* = 39; 138 papillae) were included. Compared with saline, HA reduced the surface area of black triangles (SABT) (MD: −43.0 mm^2^), increased papilla height (MD 0.22 mm), and improved patient satisfaction (MD: 17 VAS points). However, comparisons with active regenerative comparators showed no statistically significant differences. Quantitative synthesis was limited by very high heterogeneity (*I*^2^ up to 97%), small sample sizes, and methodological concerns; all included trials were judged to have *some concerns* regarding risk of bias.

**Discussion:**

Hyaluronic acid offers short-term esthetic improvements over placebo, but evidence certainty is low to moderate, and no clear superiority over other regenerative approaches was demonstrated. Substantial heterogeneity and limited methodological robustness limit the confidence in quantitative conclusions.

**Conclusions:**

Injectable HA may improve interdental papilla esthetics in the short term compared with placebo; however, its clinical advantage over other regenerative materials remains uncertain. Given the high heterogeneity, small sample sizes, and risk of bias, larger, well-designed randomized trials with standardized protocols and longer follow-up are required before firm clinical recommendations can be made.

**Systematic Review Registration:**

https://www.crd.york.ac.uk/PROSPERO/view/CRD420251051629, identifier CRD420251051629.

## 1 Introduction

Black triangles, due to interdental papillary loss, exacerbate aesthetic concerns and impair phonetics, food control, and effective plaque removal, thereby reducing patient satisfaction and increasing the risk of root caries. Loss of interdental papillary height has been systematically classified by Nordland and Tarnow using a clinically oriented and reproducible system based on well-defined anatomical landmarks. This classification categorizes papillary loss according to the position of the papilla tip relative to the interdental contact point, the interproximal cemento-enamel junction (CEJ), and the facial CEJ. Papillae are classified as normal when the embrasure is completely filled, while increasing degrees of papillary loss are designated as Class I, II, or III, reflecting progressive apical displacement of the papilla tip (1).

Existing techniques for papilla reconstruction or augmentation include surgical and non-surgical approaches. Surgical methods such as the double papilla repositioned flap and vertical interproximal tunneling combined with connective tissue grafts can achieve substantial papilla fill but are technique-sensitive and may cause appreciable morbidity and postoperative discomfort (2–4). Non-surgical options—including injectable biomaterials like platelet-rich fibrin and hyaluronic acid—offer minimally invasive alternatives with reduced patient discomfort; however, outcomes are variable, and long-term stability data remain sparse (5–8). Overall, the predictability of both surgical and non-surgical papilla augmentation remains inconsistent, underscoring the need for standardized protocols and robust comparative trials (9–11).

Hyaluronic acid (HA) is a naturally occurring glycosaminoglycan in the extracellular matrix, where its strong hydrophilicity maintains tissue hydration and its high biocompatibility minimizes immunogenic responses (12, 13). In soft-tissue contexts, HA modulates inflammation by interacting with cytokines and growth factors, enhances angiogenesis, and supports cell proliferation and migration properties that underpin its regenerative potential in periodontal tissues (13). Clinically, HA, when used as an adjunct to non-surgical periodontal therapy, reduces bleeding on probing and improves clinical attachment. Furthermore, HA-based gels and hydrogels used in mucogingival surgery accelerate wound healing, augment the gingival biotype, and enhance root coverage with minimal postoperative discomfort (14, 15). More recently, minimally invasive HA injections have been explored for interdental papilla augmentation, with pilot studies reporting moderate to high papilla fill gains at three to six months and high patient satisfaction, accompanied by few adverse events (16, 17).

Despite these promising individual reports, no systematic review has yet directly compared HA injections with established surgical methods (e.g., connective tissue grafts, tunnelling techniques) or other non-surgical methods (e.g., platelet-rich fibrin) for interdental papilla augmentation in sites with preserved interdental attachment (18). Existing evidence is primarily derived from small case series and pilot trials with heterogeneous methodologies, short follow-up periods, and a lack of standardized outcome measures, leaving uncertainty about the long-term durability and relative efficacy of HA-mediated papilla fill (16, 19). Furthermore, although reported complications are minimal, the safety profile of HA in this specific application has not been systematically evaluated. These gaps underscore the need for rigorously designed comparative trials and meta-analyses to establish optimal HA formulations, dosing regimens, and sustained clinical benefits in interdental papilla augmentation.

The central review question is framed using the PICOS framework: In adults (≥18 years), clinically evident interdental papilla deficiency or reduced papilla fill in the absence of interdental attachment loss (P), does injectable hyaluronic acid used alone or as an adjunct (I) improve interdental papilla augmentation and black-triangle reduction outcomes compared to other surgical techniques (e.g., connective tissue grafts, tunneling) or non-surgical approaches (e.g., collagen injections, platelet-rich fibrin, no treatment) (C)? The primary outcome is the Surface area of the black triangle (SABT) or papilla fill assessed by validated clinical indices, with secondary outcomes including esthetic satisfaction, soft-tissue thickness/volume gains, incidence of complications, stability over time, and need for retreatment.

The purpose of this systematic review is to rigorously evaluate and compare the clinical effectiveness, esthetic benefits, durability, and safety profile of hyaluronic acid vs. established papilla reconstruction methods in adults with Interdental papillary loss.

## 2 Material and methods

### 2.1 Protocol and registration

PROSPERO registration number CRD420251051629 (Date of registration: May 14, 2025).

### 2.2 Eligibility criteria

Only peer-reviewed original research articles published in refereed journals were eligible. Grey literature sources (e.g., books, dissertations, monographs, and textbooks) were not included in the final synthesis due to a lack of peer review and insufficient comparative clinical data.

We included studies of adults (≥18 years) diagnosed with clinically evident interdental papilla deficiency or reduced papilla fill (without interdental attachment loss) who underwent interdental papilla augmentation or reconstruction with injectable hyaluronic acid (HA), either alone or as an adjunct. Comparators comprised any other surgical (e.g., connective tissue graft, tunneling, envelope techniques) or non-surgical (e.g., collagen matrix, platelet-rich fibrin, no treatment) intervention.

Eligible study designs were randomized controlled trials and split-mouth randomized controlled trials. Observational designs (prospective or retrospective cohorts) were considered at the screening stage but were ultimately excluded because they did not meet the predefined criteria for comparative randomized evidence. We excluded animal or *in vitro* studies, case reports/series, narrative reviews, letters, editorials, PhD theses, and studies without a defined comparator group.

### 2.3 Information sources and search strategy

MEDLINE/PubMed and EMBASE were searched from inception through May 2025. The electronic strategy combined free-text terms and MeSH headings for each PICOS element:

**Population:** (“interdental papilla loss” OR “black triangle*” OR “esthetic zone*” OR “anterior teeth” OR “papilla deficienc*” OR “soft tissue loss*” OR “anterior tooth”) OR MeSH (“Gingival Recession”[Mesh] OR “Tooth, Anterior”[Mesh] OR “Periodontitis”[Mesh])
**Intervention:** (“hyaluronic acid” OR “HA gel*” OR “injectable HA” OR “hyaluronan” OR “hyaluronate” OR “dermal filler*”) OR MeSH (“Hyaluronic Acid”[Mesh] OR “Injections”[Mesh])
**Comparator:** (“connective tissue graft*” OR “papilla reconstruction surger*” OR “platelet-rich fibrin*” OR “collagen matrix” OR “tunneling technique*” OR “envelope technique*” OR “no treatment” OR “usual care”) OR MeSH (“Gingival Grafting”[Mesh] OR “Platelet-Rich Plasma”[Mesh] OR “Collagen”[Mesh] OR “Placebos”[Mesh])
**Outcomes:** (“papilla height” OR “papilla fill” OR “black triangle reduction” OR “esthetic outcome*” OR “patient satisfaction”) OR MeSH (“Treatment Outcome”[Mesh] OR “Esthetics, Dental”[Mesh] OR “Patient Satisfaction”[Mesh])

### 2.4 Study selection and data extraction

Two reviewers independently screened titles/abstracts and assessed full texts, resolving discrepancies through discussion or consultation with a third reviewer. Data were extracted using a piloted form to capture study details, participant demographics, interventions, outcomes (primary: papilla fill indices; secondary: esthetic satisfaction, tissue metrics, complications, and stability), follow-up, effect sizes, and funding/conflicts of interest; authors were contacted for missing data when necessary.

### 2.5 Risk of bias assessment

The risk of bias in randomized trials was evaluated using the Cochrane RoB 2 tool. Two reviewers independently performed assessments; disagreements were resolved by consensus. Given the small number of included trials (*n* = 3), we did not conduct formal assessments of publication bias, as these methods typically require at least 10 studies to yield reliable results.

### 2.6 Data synthesis and statistical analysis

Where ≥2 studies reported comparable outcomes, fixed-effects meta-analyses were performed for continuous (MD/SMD) and dichotomous (RR/OR) outcomes, along with 95% CIs. Given the small number of included studies and anticipated clinical and methodological heterogeneity, the quantitative synthesis was undertaken cautiously, primarily for descriptive purposes. Heterogeneity was assessed using *I*^2^; values >75% were considered indicative of considerable heterogeneity. When heterogeneity was substantial, pooled estimates were interpreted with caution and not used to draw definitive conclusions. Planned subgroup, sensitivity, and meta-regression analyses (requiring ≥10 studies) were not conducted due to the limited number of eligible trials. In such cases, results were synthesized using structured narrative summaries emphasizing individual study findings rather than pooled effects. Because outcome definitions (percentage change vs. absolute area), comparators (placebo vs. active biomaterials), and units of analysis differed substantially across trials, pooled estimates were interpreted cautiously and used only to explore consistency of direction rather than magnitude of effect.

### 2.7 PRISMA

This study adheres to the Preferred Reporting Items for Systematic Reviews and Meta-Analyses (PRISMA) guidelines.

## 3 Results

### 3.1 Study selection

**Figure 1:**
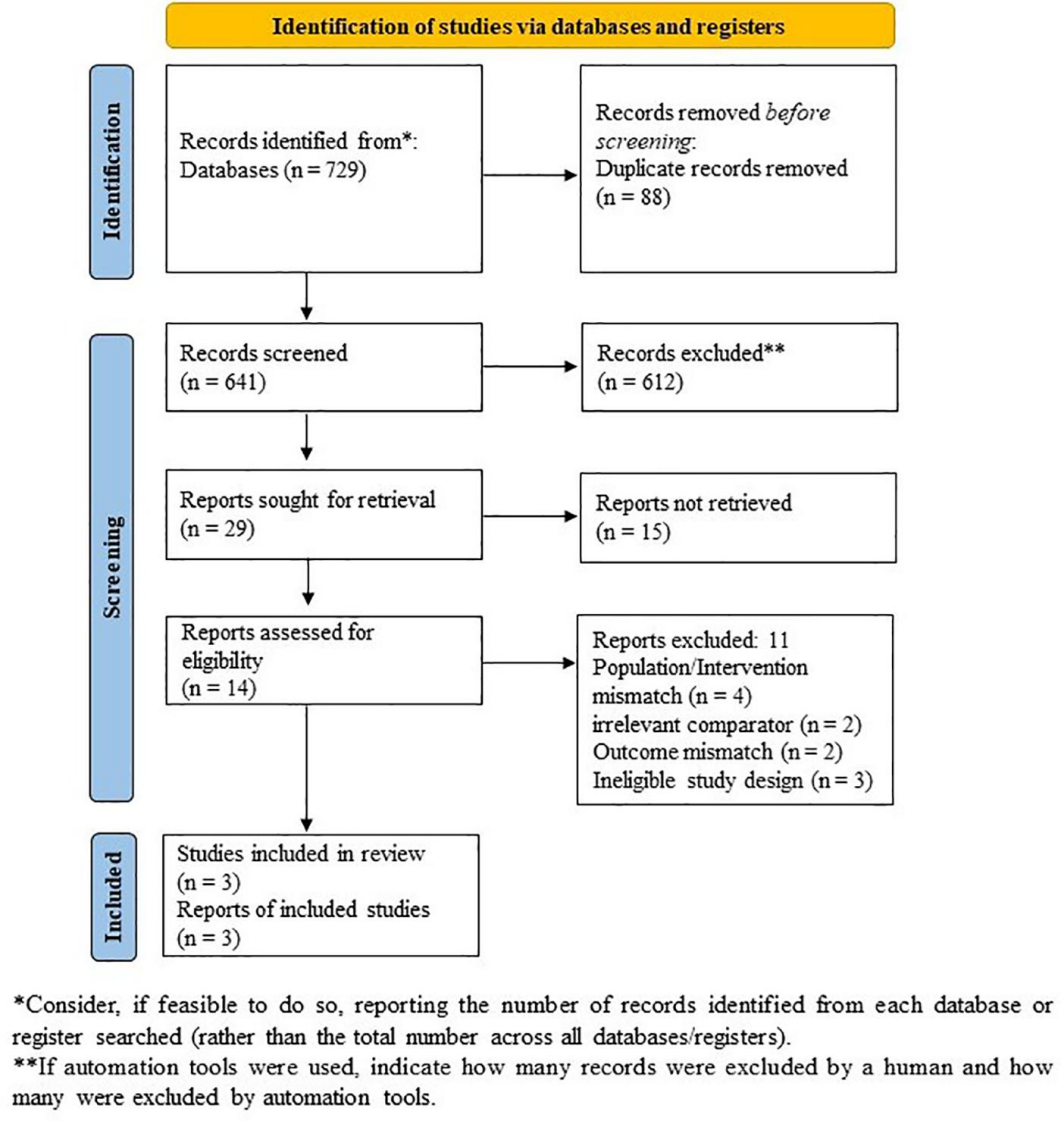
PRISMA flow diagram of study identification, screening, eligibility, and inclusion.

A total of 729 records were identified through database searches; 88 duplicates were removed, leaving 641 unique records for title/abstract screening. Of these, 612 were excluded because they did not meet the PICOS criteria (i.e., incorrect population, intervention, comparator, outcome, or study design). Twenty-nine full-text articles were sought, but 15 were unavailable because the studies were ongoing. Fourteen full-texts were assessed for eligibility; 11 were excluded because they failed to meet our predefined criteria (i.e., incorrect population/intervention/comparator). The remaining three RCTs were included in the synthesis (Figure 1).

Table 1 presents the 11 reports (19–29) excluded at full-text assessment, with the primary reason for exclusion and explanatory details for each.

**Table 1:** Excluded full-text reports after eligibility assessment (*n* = 16), with publication year, primary reason for exclusion, and explanatory details.

SN	Title	Year	Reason for exclusion	Explanation
1	A novel injectable platelet-rich fibrin reinforced papilla reconstruction technique	2022	Ineligible study design, Intervention mismatch, No relevant comparator	Case report/series (non-comparative); lacks control group or randomization. Focuses on injectable PRF, not hyaluronic acid (HA). No comparison to HA, connective tissue grafts, or other techniques.
2	A Three-Month Clinical Trial on the Efficacy of Hyaluronic Acid Adjunctive Non-Surgical Therapy for Periodontitis in Patients with Type 2 Diabetes Mellitus	2024	Population mismatch Outcome mismatch Contextual irrelevance	Focuses on periodontitis in diabetic patients, not papilla reconstruction. Evaluates periodontal parameters (BOP, CAL, PD) but not papilla fill, black triangle reduction, or esthetic outcomes. Study addresses systemic inflammation in diabetes, not localized papilla defects.
3	A tissue-engineered biocomplex for periodontal reconstruction. A proof-of-principle randomized clinical study	2021	Irrelevant Population/Intervention	Focuses on intrabony defects and stem-cell therapy, not papilla reconstruction or hyaluronic acid.
4	Comparison of Amnion Membrane and Hyaluronic Acid in Gingival Recession Coverage and Gain in Clinical Attachment Level following Coronally Advanced Flap Procedure—A Clinical Study	2023	Outcome Mismatch	Evaluates root coverage (gingival recession) and CAL gain, not interdental papilla reconstruction or black triangle reduction.
5	Effect of Hyaluronic Acid Mucoadhesives on Palatal Wound Healing and Postoperative Discomfort in Free Gingival Graft Surgery: A Clinical Trial	2024	Population/Intervention mismatch Outcome mismatch Surgical context irrelevant	Focuses on palatal wound healing post-FGG surgery, not papilla reconstruction. Assesses epithelialization and pain, not papilla fill or black triangle reduction. Examines donor site healing, not interdental papilla regeneration techniques.
6	Effects of Platelet-Rich Plasma Injections on Periodontal Health During Accelerated Orthodontic Tooth Movement	2024	Population/Intervention mismatch Outcome mismatch Intervention irrelevant	Focuses on orthodontic tooth movement, not papilla reconstruction. Evaluates orthodontic-periodontal interactions (PPD, CAL, BOP), not papilla-specific outcomes. Uses PRP, not HA or other papilla reconstruction techniques.
7	Evaluation of the Hyaluronic Acid Versus the Injectable Platelet-Rich Fibrin in the Management of the Thin Gingival Phenotype. a Split-Mouth Randomized Controlled Clinical Trial	2022	Outcome Mismatch	The study focuses on gingival thickness (GT) and keratinized tissue width (KTW) but does not evaluate papilla reconstruction, black triangle reduction, or esthetic outcomes.
8	Hyaluronic acid injection to restore the lost interproximal papilla. a systematic review.	2022	Secondary Literature	The study is a systematic review (not primary research) and thus excluded for data synthesis, though it may inform background/discussion.
9	Stability of biomaterials used in adjunct to coronally advanced flap: A systematic review and network meta-analysis	2022	Secondary literature, Systematic review, Irrelevant Population & Intervention No HA Comparator Outcomes Not Aligned Study Design Mismatch	The study focuses on root coverage procedures (e.g., connective tissue grafts, coronally advanced flaps) for gingival recession (Miller Class I/II), not papilla reconstruction or hyaluronic acid (HA) use.
10	The association between radiographic embrasure morphology and interdental papilla reconstruction using injectable hyaluronic acid gel	2016	No comparator described	Although the study focuses on injectable HA for interdental papilla reconstruction in the upper anterior esthetic zone and uses rigorous methods, no control or comparator group was included.
11	Use of different concentrations of hyaluronic acid in interdental papillary deficiency treatment: A clinical study	2019	No comparator group	The study compared different HA concentrations but lacked a control (e.g., placebo, CTG, or other active treatment).

### 3.2 Study characteristics

Across three randomized controlled trials encompassing 8–24 adult patients (30–84 interdental papilla sites) with Interdental papillary loss, injectable hyaluronic acid (HA) gels (Restylane-Lidocaine, Revofil, HyaDENT BG, Qi Sheng) were administered in two to three sessions over 3–6 weeks and compared against saline placebo, autologous albumin-PRF, or injectable PRF. Abdelraouf et al. (30) reported a 45% reduction in SABT and significantly higher patient satisfaction with HA compared with saline at 6 months. Vadiati Saberi et al. (18) found that both HA and Alb-PRF achieved comparable 64%–66% reductions in the SABT and similar aesthetic satisfaction at six months. Ni et al. (7) observed a 0.28 mm papilla height increase with HA at 12 months—mirroring saline—and *in vitro* acceleration of fibroblast proliferation. Overall, HA injections showed promise for papilla augmentation and esthetic improvement, though small sample sizes, brief follow-up, and mixed comparator efficacy highlight the need for larger, long-term studies.

The key characteristics of the three included randomized controlled trials are summarized in Table 2.

**Table 2:** Characteristics of included studies.

SN	Study title	Author & year	Study design	Population	Intervention	Comparator	Outcomes measured	Key findings	Limitations
1	Assessment of Hyaluronic Acid Gel Injection in the Reconstruction of Interdental Papilla: A Randomized Clinical Trial	Abdelraouf et al. (2019)	RCT (double-blinded, placebo-controlled)	10 patients (21–47 years) 36 deficient papillae (Nordland & Tarnow Class I/II) CP-BC ≤7 mm	HA gel (Restylane-Lidocaine) 3 injections (baseline, 3, 6 weeks)	Saline injection (placebo)	Papilla height (PT-CP distance) Black triangle surface area (SABT) Patient satisfaction (VAS)	HA group: Significant reduction in SABT (45% at 6mo) and PT-CP distance vs. placebo Higher patient satisfaction (VAS: 45 vs. 27.86)	Small sample (8 patients, 30 papillae analyzed) Short follow-up (6mo) Incomplete papilla fill in HA group No long-term stability data
2	Comparison of the Effect of Albumin with Platelet-Rich Fibrin (Alb-PRF) Gel and Hyaluronic Acid Gel Injection on Interdental Papilla Reconstruction	Saberi et al. (2024)	RCT (parallel-group)	10 patients (24–63 years) 46 deficient papillae (Nordland & Tarnow Class I/II) CP-BC ≤7 mm	HA gel (Revofil) 2 injections (21-day interval) Alb-PRF gel (autologous)	HA gel vs. Alb-PRF gel	Black triangle surface area (SABT) Patient satisfaction (VAS) Plaque index (Löe)	Both groups: Significant SABT reduction (HA: 64%, Alb-PRF: 66% at 6mo) Comparable patient satisfaction (HA: 65.24, Alb-PRF: 60.00) No significant inter-group differences	Small sample (10 patients). Age imbalance (Alb-PRF group older). Qualitative plaque index (limited sensitivity). Short follow-up (6mo)
3	Hyaluronic Acid vs. Physiological Saline for Enlarging Deficient Gingival Papillae: A Randomized Controlled Clinical Trial and *in vitro* Study	Ni et al. (2021)	RCT (split-mouth) + *in vitro*	24 patients (28–63 years) 68 deficient papillae (Nordland & Tarnow Class I/II) Maxillary anterior teeth	HA gel (Qi Sheng) 3 injections (baseline, 3, 6 weeks)	Saline injection	Papilla height SABT Fibroblast proliferation/migration (*in vitro*)	HA group: Papilla height increased by 0.28 mm at 12mo (*P* < 0.05). Saline group: Similar improvement at 12mo (0.278 mm) HA accelerated fibroblast proliferation/migration *in vitro*	No significant superiority of HA over saline Predominantly female cohort (19/21) Lack of 3D measurements Underpowered inter-group comparison (64% power)

### 3.3 Risk of bias within studies

The heat map presents risk-of-bias ratings by domain and overall, with green for “Low risk” and yellow for “Some concerns.” All three included RCTs were judged to carry an overall risk of “some concerns” using the Cochrane RoB 2 tool. Abdelraouf et al. (30) demonstrated a low risk in the randomization, deviations, and measurement domains; however, they raised some concerns due to missing outcome data and unclear pre-specification of analyses. Vadiati Saberi et al. (18) raised concerns regarding both the randomization process (lack of allocation concealment details) and deviations from intended interventions (lack of blinding), with a low risk of missing data and selection bias in the reported results. Ni et al. (7) was low risk in all domains except randomization, where inadequate detail on allocation concealment raised some concerns.

### 3.4 Results of individual studies

Across the three randomized trials, hyaluronic acid (HA) injections demonstrated clear benefits over placebo but yielded modest or non-significant advantages when compared with other active interventions. In the placebo-controlled trial, Abdelraouf et al. (30) reported a 45% mean reduction in black-triangle surface area (MD: −43.0 mm^2^; 95% CI, −58.2 to −27.8) and a −0.22-mm increase in papilla height (95% CI, −0.36 to −0.08) at six months, alongside a 17-point higher patient satisfaction score on a 0–100 VAS (95% CI, 8.1–26.2). Vadiati Saberi et al. (18) found virtually identical SABT reductions with HA and albumin-PRF (64% vs. 66%; MD: −0.004 mm^2^; 95% CI, −0.014 to 0.006) and no significant difference in satisfaction (MD 5.2 VAS points; 95% CI, −9.9 to 20.3). Finally, Ni et al. (7) observed negligible differences between HA and saline in papilla height at six months (MD: 0.06 mm; 95% CI, −0.12 to 0.25) and twelve months (MD: 0.002 mm; 95% CI, −0.21 to 0.21), as well as similar reductions in SABT (7). These data indicate that HA injections reliably outperform inert placebo but offer only marginal benefits over other regenerative materials. Figure 3 illustrates the forest plot of mean differences in black-triangle surface area (SABT) comparing hyaluronic acid gel vs. saline placebo across two randomized trials. As shown, the pooled MD was −0.12 mm^2^ (95% CI, −0.31 to 0.08; *Z* = 1.17, *p* = 0.24) under a fixed-effects model, with high heterogeneity (*χ*^2^ = 30.63, *df* = 1, *p* < 0.00001; *I*^2^ = 97%).

Figure 3. Forest plot of mean differences in black-triangle surface area (SABT, mm^2^) comparing hyaluronic acid gel vs. saline placebo across two randomized trials. Squares represent individual study mean differences (MD) with 95% confidence intervals (CIs) and weights under a fixed-effects model; the diamond shows the pooled MD (−0.12 mm^2^; 95% CI, −0.31 to 0.08; *Z* = 1.17, *P* = 0.24). Heterogeneity was high (*χ*^2^ = 30.63, *df* = 1, *P* < 0.00001; *I*^2^ = 97%).

### 3.5 Synthesis of results

An exploratory meta-analysis of SABT from two RCTs (18, 30; total *n* = 96 papillae) showed a pooled mean difference of −0.12 mm^2^ (95% CI, −0.31 to 0.08; *Z* = 1.17, *p* = 0.24), indicating no statistically significant overall effect (Figure 3). However, this analysis demonstrated very high heterogeneity (*I*^2^ = 97%), likely reflecting differences in comparators (saline placebo vs. Alb-PRF), study designs, and outcome measurement methods. The large mean difference observed in the placebo-controlled trial (MD: −43.0 mm^2^) and the near-null pooled estimate from meta-analysis (MD: −0.12 mm^2^) reflect fundamental methodological differences in measurement scale, comparator type, and analytical unit, rather than contradictory treatment effects. Abdelraouf et al. (30) reported percentage-based change in SABT from baseline within a single parallel-group trial comparing hyaluronic acid with inert saline placebo. In contrast, the meta-analysis pooled absolute end-point differences (mm^2^) across trials with heterogeneous comparators, including an active regenerative control (albumin-PRF). It was conducted at the papilla level rather than the patient level. Consequently, effect sizes were not directly comparable, and the pooled estimate should be interpreted as exploratory only, particularly given the extreme heterogeneity (*I*^2^ = 97%). Meta-analysis of patient satisfaction and secondary outcomes (e.g., gingival thickness, keratinized tissue width, gingival index, bleeding on probing, and probing depth) was not feasible due to insufficient availability of comparable data across studies; therefore, these outcomes were summarized narratively. HA consistently outperformed the saline placebo but showed no clear or consistent advantage over Alb-PRF or i-PRF in split-mouth trials or active-comparator designs.

### 3.6 Risk of bias across studies

**Figure 2:**
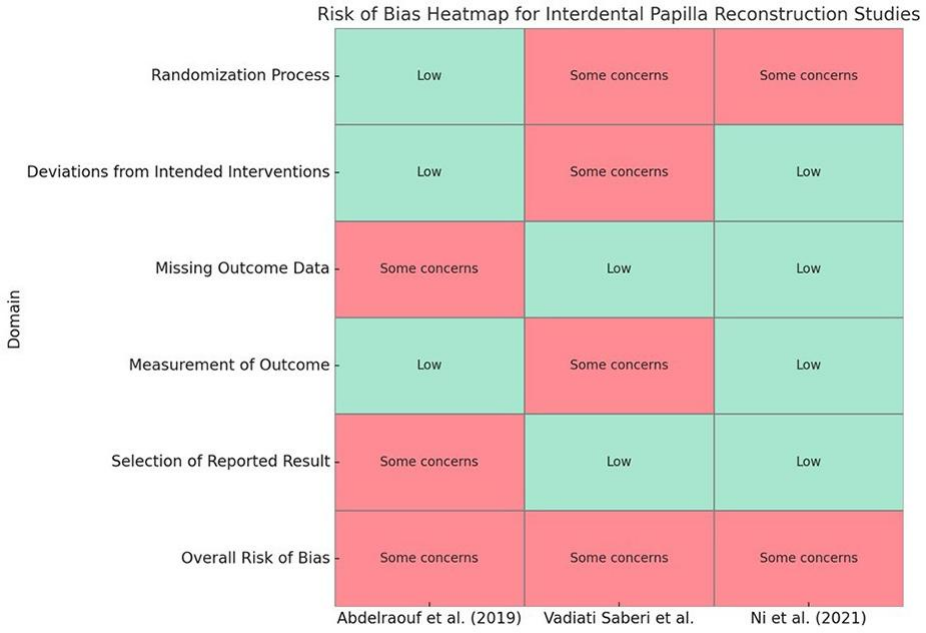
Heat map of risk of bias (RoB 2) across five domains and overall.

Across the three included RCTs, formal assessment of publication bias (e.g., funnel plots or Egger's test) was not performed because fewer than ten studies were available. When we aggregate RoB 2 domain ratings across studies (Figure 2), the most frequent area of concern was the randomization process (some concerns in 2/3 trials), followed by deviations from intended interventions, missing outcome data, measurement of outcome, and selection of reported results (each with some concerns in 1/3 trials). Consequently, every trial carried an overall judgment of “some concerns,” reflecting isolated methodological or reporting issues but no pervasive high-risk domains across the body of evidence.

**Figure 3:**
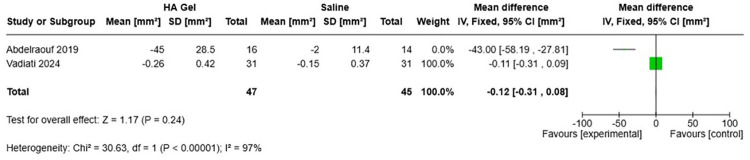
Forest plot of mean differences in black-triangle surface area (SABT, mm^2^).

## 4 Discussion

Hyaluronic acid (HA) injections produced a significant reduction in surface area of black triangles (SABT) vs. saline placebo (MD: −43.0 mm^2^; 95% CI, −58.2 to −27.8); however, the certainty of this evidence should be considered *low to moderate* due to methodological limitations, including missing outcome data and incomplete reporting of allocation concealment. In comparisons with active regenerative controls such as albumin-PRF, HA showed no significant advantage (MD: −0.004 mm^2^; 95% CI, −0.014 to 0.006), and the certainty of evidence for these comparisons is *low* given unclear allocation concealment, lack of participant or provider blinding, and small sample sizes. In a single placebo-controlled RCT, hyaluronic acid (HA) injections were associated with a large reduction in SABT mean difference (MD) −43.0 mm^2^ (95% CI, −58.2 to −27.8) vs. saline placebo yet show no significant advantage over albumin-PRF gel (MD: −0.004 mm^2^; 95% CI, −0.014 to 0.006), and the certainty of evidence for these comparisons is low, given unclear allocation concealment, lack of participant or provider blinding, and small sample sizes. Importantly, the pronounced placebo-controlled effect was not reproduced when data were pooled across heterogeneous trials using absolute end-point measures and active comparators, underscoring the need for cautious interpretation of pooled estimates (18.31).

For papilla height, a modest increase of 0.22 mm (95% CI, 0.08–0.36) with HA vs. saline was observed, whereas split-mouth data revealed a negligible pooled MD of −0.05 mm (95% CI, −0.19 to 0.09). As all included trials were judged as having “some concerns” under RoB 2—mainly due to unclear allocation concealment and incomplete blinding—the certainty of evidence for papilla height outcomes was considered low, further limited by the small number of studies, wide confidence intervals, and heterogeneity in comparators (7, 30).

Patient-reported esthetic satisfaction was 17 points higher on a 100-point VAS with HA vs. saline (95% CI, 8–26), but did not differ significantly from albumin-PRF (MD = 5.2; 95% CI, −9.9 to 20.3). Interpretation of these patient-reported outcomes is constrained by small sample sizes and incomplete blinding in some trials, resulting in *low certainty* evidence for subjective outcomes (18, 30).

Multiple systematic reviews are broadly consistent with the short-term esthetic benefits observed in the present review but underscore the need for more rigorous trials (19, 31). Ficho et al. (31) pooled data from case series and controlled trials to report a weighted-average papilla fill of 77.4% at 6 months post-injection, characterizing HA as a stable and practical non-surgical approach to black-triangle reduction.

Patel et al. (12) examined 45 studies, including seven RCTs. They noted consistent promise when HA was combined with surgical or orthodontic techniques, but substantial clinical and methodological heterogeneity precluded meta-analysis or firm guideline recommendations. Castro-Calderón et al. (19) similarly found positive papilla re-establishment but cautioned that inconsistent injection protocols and potential postoperative complications limit clinical uptake. Alsharif and Aljahdali (32) reviewed 13 prospective studies and 2 RCTs, deeming the overall study quality acceptable; however, the methodological diversity prevented quantitative synthesis, supporting HA as a practical, minimally invasive option for anterior papilla enhancement rather than a definitive standard of care.

In the context of surgical root coverage, Manfredini et al. (33) and Kalimeri et al. (34) both evaluated HA as an adjunct to coronally advanced flap procedures and found only marginal or no meaningful improvement in root coverage or long-term stability compared to flap surgery alone. Collectively, these findings indicate that HA injections may provide short-term esthetic benefits over placebo, but the overall certainty of evidence ranges from low to moderate, and confidence in comparative effectiveness against other regenerative materials remains limited due to consistent risk-of-bias concerns across trials. Overall, these reviews align with our findings: HA injections reliably outperform placebo for short-term papilla fill but offer limited advantages over established surgical and non-surgical modalities. Accordingly, future RCTs should prioritize standardized HA formulations, validated papilla indices, larger patient cohorts, extended follow-up, and cost-effectiveness analyses to clarify HA's definitive role in periodontal esthetic therapy.

For clinicians, HA injections provide a minimally invasive, office-based alternative to surgical grafting for interdental papilla augmentation, offering predictable short-term aesthetic improvement rather than confirmed long-term stability (35). In a placebo-controlled RCT, Abdelraouf et al. (30) demonstrated a 45% reduction in SABT vs. saline. However, HA should not be considered a replacement for connective tissue grafting, as head-to-head comparisons reveal no long-term advantage in root coverage or papilla stability when added to coronally advanced flaps (18, 33).

From the patient perspective, HA injections are associated with high satisfaction and minimal morbidity: mean VAS scores improved by 17 points over placebo (30), and no serious adverse events have been reported. This favorable short-term risk–benefit profile enhances HA use in patients seeking esthetic improvements without surgical intervention, particularly for localized papilla deficiencies.

Policy-makers and guideline developers should recognize that, despite promising short-term outcomes, the current evidence base remains insufficient for formal recommendations, being constrained by small sample sizes (10–24 patients), heterogeneous protocols, short follow-up (3–12 months), and an absence of health-economic evaluations (18). Formal recommendations and reimbursement decisions will require larger, multicenter RCTs with standardized HA formulations, validated papilla indices, extended follow-up durations, and cost-effectiveness analyses to confirm the durability, clinical utility, and value of HA injections in periodontal esthetic therapy.

Several limitations temper the interpretation of our findings. At the study level, the included RCTs exhibited methodological concerns, missing outcome data for up to 17% of papillae (30), unclear allocation concealment in two trials (7, 18), and incomplete assessor blinding in all the three (7, 18, 30) in addition to small cohorts (10–24 patients) and brief follow-up (3–12 months) that limit long-term inferences. At the outcome level, heterogeneous measurement methods (probe-based distances vs. image-analysis planimetry) and the absence of standardized, validated papilla indices reduce comparability. At the same time, no trial reported patient-centered quality-of-life or cost-utility outcomes. At the review level, 15 potentially eligible studies remained ongoing and unavailable for inclusion, formal publication-bias assessment was precluded by the small number of trials, and restricting our search to English-language studies in three databases may have omitted relevant non-English or grey-literature sources.

## 5 Conclusions

Hyaluronic acid (HA) injections may provide clinically meaningful short-term improvements in interdental papilla fill and patient-reported esthetics compared to a placebo (30). However, the certainty of this evidence is low to moderate, as all included randomized trials were judged to have some concerns under the RoB 2 tool, primarily related to incomplete blinding, unclear allocation concealment, and missing outcome data. Comparisons with other regenerative approaches, such as albumin-PRF or injectable PRF, demonstrated no consistent or clinically relevant advantage for HA, and the certainty of evidence for these active-comparator comparisons is low.

Given the small sample sizes, heterogeneous HA formulations and injection protocols, short follow-up durations, and methodological limitations of the available trials, HA injections cannot currently be recommended as a superior standalone or adjunctive technique for interdental papilla reconstruction relative to established surgical or biologically based therapies. Future research should focus on well-powered, multicenter randomized controlled trials using standardized HA products, validated papilla-specific outcome measures, longer follow-up periods to assess durability, and formal cost-effectiveness and quality-of-life evaluations to more clearly define the clinical role of HA in periodontal esthetic therapy.

## Data Availability

The original contributions presented in the study are included in the article/Supplementary Material, further inquiries can be directed to the corresponding author.
